# A Rare Presentation of Transfusional Hemochromatosis: Hypogonadotropic Hypogonadism

**DOI:** 10.1155/2015/493091

**Published:** 2015-07-21

**Authors:** Rifki Ucler, Erdal Kara, Murat Atmaca, Sehmus Olmez, Murat Alay, Yaren Dirik, Aydin Bora

**Affiliations:** ^1^Department of Endocrinology and Metabolism, Medical Faculty, Yuzuncu Yil University, 65080 Van, Turkey; ^2^Department of Hematology, Medical Faculty, Yuzuncu Yil University, 65080 Van, Turkey; ^3^Department of Gastroenterology, Medical Faculty, Yuzuncu Yil University, 65080 Van, Turkey; ^4^Department of Internal Medicine, Medical Faculty, Yuzuncu Yil University, 65080 Van, Turkey; ^5^Department of Radiology, Medical Faculty, Yuzuncu Yil University, 65080 Van, Turkey

## Abstract

Hemochromatosis is a disease caused by extraordinary iron deposition in parenchymal cells leading to cellular damage and organ dysfunction. *β*-thalassemia major is one of the causes of secondary hemochromatosis due to regular transfusional treatment for maintaining adequate levels of hemoglobin. Hypogonadism is one of the potential complications of hemochromatosis, usually seen in patients with a severe iron overload, and it shows an association with diabetes and cirrhosis in adult patients. We describe a patient with mild transfusional hemochromatosis due to *β*-thalassemia major, presenting with central hypogonadism in the absence of cirrhosis or diabetes. Our case showed an atypical presentation with hypogonadotropic hypogonadism without severe hyperferritinemia, cirrhosis, or diabetes. With this case, we aim to raise awareness of hypogonadotropic hypogonadism in patients with intensive transfused thalassemia major even if not severe hemochromatosis so that hypogonadism related complications, such as osteoporosis, anergia, weakness, sexual dysfunction, and infertility, could be more effectively managed in these patients.

## 1. Introduction

Hemochromatosis is a disease caused by extraordinary iron deposition in parenchymal cells leading to cellular damage and organ dysfunction. The disorder has two classifications: genetic (or primary) hemochromatosis and secondary (or acquired) hemochromatosis [1]. *β*-thalassemia major is one of the causes of secondary hemochromatosis with adoption of an intensive transfusional regimen to maintain adequate levels of hemoglobin. In these patients, increased absorption of iron from the gastrointestinal tract as a consequence of ineffective erythropoiesis and chronic transfusion therapy causes iron accumulation, which can be decreased by adequate iron chelation therapy [2–4]. When serum levels are elevated, iron is preliminarily deposited in the reticuloendothelial cells; however, when their capacity is saturated, the excess iron is deposited in parenchymal cells of the liver, spleen, pancreas, and bone marrow in a crystalline form as ferritin and hemosiderin [5]. Thus, cirrhosis and diabetes are known clinical manifestations in patients with transfusional hemochromatosis. Moreover, as iron storage continues to increase, there is deposition in the skin, heart, gonads, and endocrine glands [1, 6, 7].

Hypogonadism, secondary to pituitary dysfunction, is thought to be due to iron-induced cellular damage to pituitary gonadotrophs [6–8]. Patients without hepatic cirrhosis, diabetes mellitus, or markedly elevated serum ferritin levels are unlikely to have hypogonadism [9]. In contrast to the known situation, our case presented hypogonadotropic hypogonadism without expected organ involvement in hemochromatosis. With this case, we aim to raise awareness of hypogonadotropic hypogonadism in patients with intensive transfused thalassemia major even if not severe hemochromatosis.

## 2. Case Report

**Figure 1:**
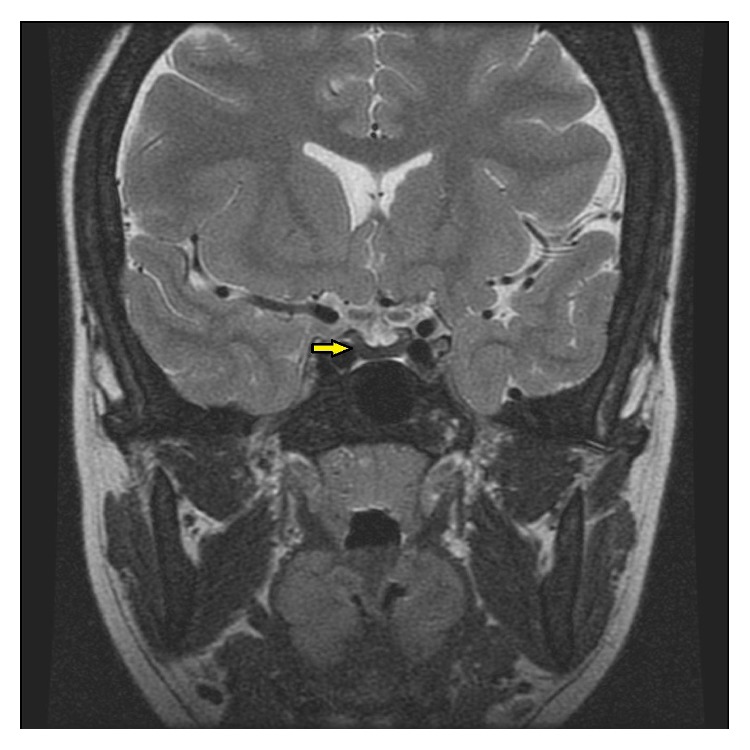
T2-weighted MRI showed decreased signal intensity of the pituitary gland, compatible with iron deposition.

A 26-year-old female patient was referred to our clinic with a 3 yr history of amenorrhea. Medical history showed a diagnosis of *β*-thalassemia major since the age of one and treatment with regular blood transfusions (once a month until the age of seven, thereafter twice a month) to maintain adequate levels of hemoglobin. She had also undergone splenectomy due to hypersplenism and massive splenomegaly at eight years old. She received iron chelation therapy with deferasirox (500 mg t.i.d.) for the last 6 years, having had irregular desferroxamine treatment before this. Her menarche was at the age of 13 years. She had a regular menstrual cycle over the next 10 years. There were no other possible causes of functional hypothalamic amenorrhea such as weight loss, eating disorders, excessive exercise, and psychosocial stress. Her blood pressure was 110/65 mmHg, she was 168 cm tall, and she weighed 53 kg. Stages of female breast and pubic hair development, according to Marshall and Tanner, were stages B-4 and P-5, respectively. There were no pathological findings except for skin hyperpigmentation on physical examination. The patient had low LH and FSH levels in association with the low estradiol levels. A bolus of 100 g synthetic LHRH was administered intravenously, and serum samples for gonadotropin measurements were drawn 0, 30, 60, 90, and 120 minutes after LHRH injection. Even after stimulation with LHRH, pituitary response was subnormal, consistent with hypogonadotropic hypogonadism. Peak levels of growth hormone and cortisol with insulin tolerance test were 11.6 ng/mL and 26.3 *μ*g/dL, respectively. Her serum ferritin was 887 ng/mL (normal range 4.6–204) and transferrin saturation was 66.4%. Other laboratory test results were normal except for the anemia and thrombocytosis (Table 1). Abdominal magnetic resonance imaging was unremarkable except for asplenia. Magnetic resonance imaging (MRI) showed decreased signal intensity of the pituitary gland on T2-weighted images (Figure 1). With these findings, the patient was accepted as isolated gonadotropin deficiency resulting from iron deposition in the pituitary gland. Additionally, bone densitometry (BMD) showed osteopenia, with a *Z* score of −1.8 in the femur and −2.1 in the spine. Combined estrogen/progesterone replacement therapy and calcium/vitamin D supplementation therapy were then prescribed for hypogonadism and osteopenia.

## 3. Discussion

Diabetes and hepatic cirrhosis are the usual complications of hemochromatosis. The main pathophysiological mechanism leading to these diseases in haemochromatosis is thought to involve beta-cell and hepatocyte dysfunction, with iron deposition directly damaging the pancreatic islets and hepatocytes [1, 8, 10]. Despite its relatively low prevalence, hypogonadism is an important complication of hemochromatosis. Other pituitary axes are generally normal in pituitary insufficiency due to hemochromatosis, indicating an affinity of iron for gonadotropic cells [2, 6–8]. Identification of hypogonadism is very crucial, since its presence may be associated with significant long-term morbidity, including osteoporosis, anergia, weakness, sexual dysfunction, and infertility. Hormone replacement therapy can significantly improve the quality of life of these patients by restoring sexual function [11].

In our case, the endocrine profile was consistent with hypogonadotropic hypogonadism, but there was no evidence of liver damage or diabetes mellitus. This situation was exceptional for hypogonadism associated with hemochromatosis, with the absence of severe hyperferritinemia, hepatic cirrhosis, or diabetes [9]. In furtherance of this association, Lu et al. [12] reported a 23-year-old man with beta-thalassemia major and transfusional hemochromatosis, which manifested as diabetic ketoacidosis and hypogonadotropic hypogonadism. Magnetic resonance imaging of the abdomen showed decreased signal intensity in the liver, spleen, and pancreas in their case. In our case, MR imaging of abdomen was unremarkable. In addition, the pituitary gland also showed heterogeneous low signal intensity in these cases like our case.

We did not perform a liver biopsy in this case, because of The American Association for the Study of Liver Disease advisory that liver biopsy is not necessary in patients less than 40 years of age in whom liver blood tests are normal and in whom serum ferritin is less than 1000 ng/mL [13]. Additionally, genetic causes of isolated hypogonadotropic hypogonadism such as KAL1, FGFR1/FGF8, PROKR2/PROK2, CHD7, or GNRH1 gene mutations [14] have not been studied because the patient had a normal puberty and regular menstruation before the amenorrhea.

Bone changes and osteoporosis may also influence the functional prognosis and quality of life of patients with hemochromatosis [15, 16]. In our case, the reduction in BMD was consistent with osteopenia. We found low vitamin D status and high parathormone (PTH) levels in our patient. The combination of estradiol deficiency, direct iron toxicity to osteoblasts, and vitamin D deficiency probably contributed to the low bone mass in our patient.

Magnetic resonance imaging can be used as a noninvasive method to provide identification of pituitary iron overload to support clinical and laboratory data in patients with transfusional hemochromatosis. The best predictor of pituitary iron overload is the detection of decreased signal intensity of the anterior lobe of the pituitary gland on T2-weighted MRI [6, 7]. T2-weighted MR images showed decreased signal intensity of the pituitary gland, compatible with iron deposition, which enabled us to rule out other causes in our patient (Figure 1).

In conclusion, our case has shown that hypogonadism is an important complication in transfusional hemochromatosis without overt signs of severe iron accumulation. Careful clinical, hormonal, and radiological assessment for hypogonadism should constitute an essential part of the evaluation of patients with thalassemia major even if not severe hemochromatosis. Additionally, T2-weighted MR images must be considered in evaluating such patients. Thus, management of hypogonadism related complications such as osteoporosis, anergia, weakness, sexual dysfunction, and infertility could be more effective in patients with hemochromatosis.

## Figures and Tables

**Table 1:** Laboratory results of the case.

		Reference range
CBC parameters		
WBC (10^3^/*μ*L)	11.8	4.8–10.8
Hb (g/dL)	8.9	12–16
HCT (%)	27.3	37–47
RBC (10^3^/*μ*L)	3.05	4.2–5.4
MCV (fL)	89.5	(80–94)
MCH (g/dL)	29.4	32–36
RDW (%)	17.4	10–20
PLT (10^3^/*μ*L)	1239	130–400

Hormonal measurements		
FSH (*μ*IU/mL)	1.39	3.03–8.08^*∗*^
LH (*μ*IU/mL)	0.75	2.39–6.6^*∗*^
Estradiol (pg/mL)	<10	21–251^*∗*^
Progesterone (mg/mL)	0.2	0–0.3^*∗*^
GH (ng/mL)	2.53	(0–8)
Somatomedin-C (ng/mL)	151	(90–271)^*∗∗*^
ACTH (pg/mL)	43.2	(0–46)
Cortisol (*μ*g/dL)	15.9	(3.7–19.4)
Prolactin (mg/mL)	9.29	(5.2–26.5)
sT3 (pg/mL)	3.64	1.71–3.71
sT4 (ng/dL)	1.21	0.7–1.48
TSH (*μ*IU/mL)	1.38	0.35–4.94
PTH (pg/mL)	95.7	15–68.3
25-OH-vitamin D (ng/mL)	11.9	15–60

Biochemical measurements		
Glucose (mg/dL)	81	65–95
Cre (mg/dL)	0.63	0.7–1.3
AST (U/L)	18	0–31
ALT (U/L)	15	0–31
GGT (U/L)	16.3	5–36
ALP (U/L)	245	0–270
T. bil (mg/dL)	2.8	0.2–1.2
D. bil (mg/dL)	0.44	0–0.5
T. prot (g/dL)	7.5	6.6–8.7
Alb (g/dL)	5	3.5–5.2
Ca (mg/dL)	9.7	8.5–10.5
Iron (*μ*g/dL)	239	37–145
Ferritin (ng/mL)	887	4.6–204
TIBC (*μ*g/dL)	357	215–480

*Dynamic tests *		
LHRH stimulation test (0, 30, 60, 90, and 120 min):		
FSH (*μ*IU/mL): 1.14–1.33–1.41–1.72		
LH (*μ*IU/mL): 0.75–0.75–0.80–0.91		
Insulin tolerance test:		
Peak GH: 11.6 ng/mL		
Peak cortisol: 26.3 *μ*g/dL		

^*∗*^For follicular phase.

^*∗∗*^For age 26–30.
